# Anti-Inflammatory Effects of Calcium Hydroxide Combined with Ellagic Acid as Pulp Capping Material: *In Vivo* Study

**DOI:** 10.1055/s-0044-1791243

**Published:** 2024-11-07

**Authors:** Annisa Fitria Sari, Intan Nirwana, Anita Yuliati, Asti Meizarini, Retno Pudji Rahayu, Retno Palupi, Michelle Fidelia Alexandra, Tarissa Balqis Nuraida, Meircurius Dwi Condro Surboyo, Khairul Anuar Shariff

**Affiliations:** 1 Dental Health Science Programme, Faculty of Dental Medicine, Universitas Airlangga Surabaya, Surabaya, Indonesia; 2 Department of Dental Materials, Faculty of Dental Medicine, Universitas Airlangga Surabaya, Surabaya, Indonesia; 3 Department of Oral Pathology and Maxillofacial, Faculty of Dental Medicine, Airlangga University, Surabaya, Indonesia; 4 Department of Dental Public Health, Faculty of Dental Medicine, Universitas Airlangga Surabaya, Surabaya, Indonesia; 5 Dental Science Programme, Faculty of Dental Medicine, Universitas Airlangga Surabaya, Surabaya, Indonesia; 6 Department of Oral Medicine, Faculty of Dental Medicine, Airlangga University, Surabaya, Indonesia; 7 School of Materials and Mineral Resource Engineering, Universiti Sains Malaysia, Penang, Malaysia

**Keywords:** calcium hydroxide, ellagic acid, pulp capping, inflammation, human health

## Abstract

**Objectives** Pulp capping is a pivotal treatment in dentistry aimed at preserving pulp vitality. While calcium hydroxide has long been considered the gold standard in pulp capping materials, its long-term use can induce chronic inflammation, ultimately leading to pulp necrosis and affecting human health. In this context, ellagic acid, a natural compound with potent anti-inflammatory properties, emerged as a promising adjunct to mitigate inflammation associated with calcium hydroxide application, thereby sustaining pulp vitality. This study aimed to investigate the inflammatory response by alterations in neutrophil, macrophage, lymphocyte, and tumor necrosis factor-α (TNF-α) expression following the treatment with a combination of calcium hydroxide and ellagic acid.

**Materials and Methods** Dental pulp perforation was made on 27 male Wistar rats on the upper first molar and then pulp capped with calcium hydroxide and ellagic acid. The pulp of the control positive group was capped with calcium hydroxide, and the control group was not capped. The teeth were then extracted after 1, 3, and 7 days posttreatment. The differences in the number of neutrophils, macrophages, lymphocytes, and TNF-α expression were analyzed using one-way analysis of variance (ANOVA) and Tukey's honestly significant difference (HSD) test.

**Results** The treatment combination of calcium hydroxide and ellagic acid showed the lowest neutrophil number and TNF-α expression compared with the other groups ( *p* < 0.05), while the macrophage and lymphocyte numbers were the highest compared with the other groups ( *p* < 0.05).

**Conclusion** The combination of calcium hydroxide and ellagic acid as a pulp capping material exhibited a dual effect on the inflammatory response in dental pulp. These findings suggest that calcium hydroxide and ellagic acid modulate the inflammatory response in a complex manner, promoting a more controlled and potentially beneficial healing process.

## Introduction

Inflammation is a critical factor in pulp capping treatments due to its direct impact on the healing and preservation of dental pulp. 1 When the dental pulp is exposed or nearly exposed due to caries or trauma, it triggers an inflammatory response. This initial inflammation is the body's natural defense mechanism to protect against infection and facilitate healing. However, excessive or prolonged inflammation and extended caries can lead to adverse outcomes such as pulp necrosis, chronic pain, and failure of the pulp capping procedure. 2 Pulp capping is a dental treatment aimed at facilitating pulp healing by using appropriate bioactive materials to promote the formation of reparative dentin. 3 Direct pulp capping is performed in cases with exposed vital pulp. 4 Currently, calcium hydroxide is employed as a lining and cement-based material in cases of exposed pulp or deep cavities, known as direct or indirect pulp capping. 5

Calcium hydroxide possesses antimicrobial properties, a high pH, and the ability to stimulate secondary dentin formation over an extended period following pulp injury. It neutralizes low pH environments and is easy to use. 6 Despite these advantages, calcium hydroxide lacks significant mechanical strength and thermal insulation capability. However, it can neutralize acids and induce secondary dentin formation. 7 Some drawbacks of calcium hydroxide include weak dentin bonding, high solubility, potential for causing internal root resorption, and chronic inflammation. 8 Studies indicate that less than 50% of direct pulp capping treatments using calcium hydroxide are successful. 5

Ellagic acid is a polyphenol compound found in fruits and nuts such as pomegranates, raspberries, strawberries, and walnuts. It possesses notable antibacterial and anti-inflammatory properties. 9 One of the anti-inflammatory properties of ellagic acid is its ability to inhibit the activity of nuclear factor kappa B (NFκB) and heat shock protein 70 (Hsp70), which are crucial points in inflammation signaling. 10 When combined with calcium hydroxide, the resulting mixture demonstrates favorable mechanical characteristics, good compressive strength, nontoxicity, and antibacterial efficacy against *Streptococcus mutans* , *Enterococcus faecalis* , and *Lactobacillus acidophilus* . 11 Combining calcium hydroxide with ellagic acid may enhance the mechanical properties and antibacterial efficacy of calcium hydroxide as a pulp capping material. 12 To substantiate this potential, *in vivo* studies are necessary to evaluate the anti-inflammatory effects of the calcium hydroxide and ellagic acid combination. The specific aim of this study is to evaluate the anti-inflammatory effect of a combination of calcium hydroxide and ellagic acid as a pulp capping material. This evaluation focuses on the material's effects on both acute and chronic phases of inflammation, as well as the expression of proteins involved in the inflammatory process, such as tumor necrosis factor-α (TNF-α), in the pulp tissue of animal models.

## Materials and Methods

### Animal

This study received ethical approval from the Faculty of Dental Medicine, Universitas Airlangga, Indonesia (No. 1424/HRECC.FODM/XII/2023). The research design was a laboratory experimental study with a posttest control group design, using 27 male Wistar rats ( *Rattus norvegicus* ), aged 12 to 16 weeks, weighing 200 to 250 g. The rats were in good health, characterized by active movement, and underwent a 1-week adaptation period.

### Calcium Hydroxide and Ellagic Acid Preparation

An ellagic acid solution was prepared by dissolving ellagic acid powder (Ellagic acid 98%, Xi'an Biof Bio-Technology Co, China) in sterile water at a ratio of 1:7 (w/w). This solution was then mixed with calcium hydroxide (Calcium hydroxide, Merck KGaA, Darmstadt, Germany) at a ratio of 1:1 (w/w). 13

### Dental Pulp Perforation Model

The pulp chamber was opened by creating a class I cavity preparation on the upper first molar using a low-speed round bur (diameter 0.8 mm) at a depth of 1.5 to 2 mm. 14 Sterile water was used for irrigation during the preparation. Once the pulp roof was reached, the pulp chamber was perforated using a dental probe (diameter 0.46 mm) with gentle pressure. The perforated pulp chamber was marked by bleeding and confirmed with a paper point. During the procedure, the animals were anesthetized with intramuscular ketamine HCl (0.2 mL/injection).

After perforation, all the animals were randomly assigned into nine group, based on the treatment type and duration of treatment. The perforated dental pulp was treated with a pulp capping material consisting of calcium hydroxide and ellagic acid paste and applied with a micro-brush. The positive control group received calcium hydroxide as the pulp capping material, while the negative control group received only sterile saline irrigation. Following the application of the pulp capping material, all cavities were filled with glass ionomer cement (Fuji II LC, GC International Corp., Tokyo, Japan). After days 1, 3, and 7 posttreatment, the animals were sacrificed, and the maxillary tissues were collected for histological examination of the upper first molars. Before sacrifice, the animal that showed failure of glass ionomer cement filled or infection like dental abscess are excluded.

### Histological Analysis

The collected tissues were embedded in paraffin and sectioned to a thickness of 5 µm. Hematoxylin and eosin staining was performed to observe and analyze inflammatory cells in the pulp tissue, including neutrophils, macrophages, and lymphocytes. Immunohistochemical staining using antibody anti-TNF-α (mouse monoclonal, Santa Cruz Biotechnology) was conducted to observe and analyze TNF-α expression in the pulp tissue. The 3,3ʹ-diaminodbenzidine (DAB) system (Universal HRP Excell Stain, Biogear, Life Science) was used as the secondary antibody. Hematoxylin 560 (Leica Biosystem) was used as counterstain. The examination of histopathology and immunohistopathology in tissue was performed in a blinded manner by a single oral pathologist using a light microscope at 400x magnification across five different fields of view.

### Statistical Analysis

Differences in the number of neutrophils, macrophages, lymphocytes, and TNF-α expression in the pulp tissue among the control, calcium hydroxide, and calcium hydroxide and ellagic acid groups were analyzed using one-way analysis of variance (ANOVA) and post hoc Tukey's honestly significant difference (HSD) test, with a significance level set at 0.05.

## Result

### Neutrophils

**Fig. 1:**
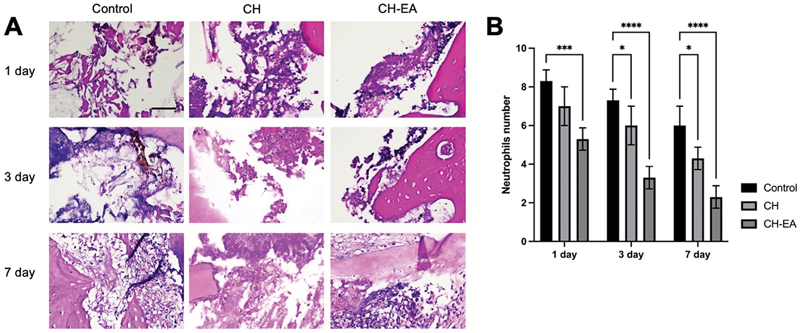
The histopathology section of dental pulp tissue with hematoxylin and eosin. ( **A** ) The neutrophils appear on the magnification of 400x. ( **B** ) The comparison of neutrophil numbers in each group. The application of calcium hydroxide and ellagic acid showed a lower number compared with control. The asterisk symbols indicate significant differences with using one-way and post hoc Tukey's honestly significant difference (HSD). * *p* < 0.05; *** *p* < 0.001; **** *p* < 0.0001. CH, calcium hydroxide; CH-EA, calcium hydroxide-ellagic acid.

Pulp capping with calcium hydroxide and ellagic acid affects acute inflammatory response by decreasing the number of neutrophils after 1, 3, and 7 days compared with pulp capping with calcium hydroxide alone or the control group ( *p* < 0.05; Fig. 1 ).

### Macrophages

**Fig. 2:**
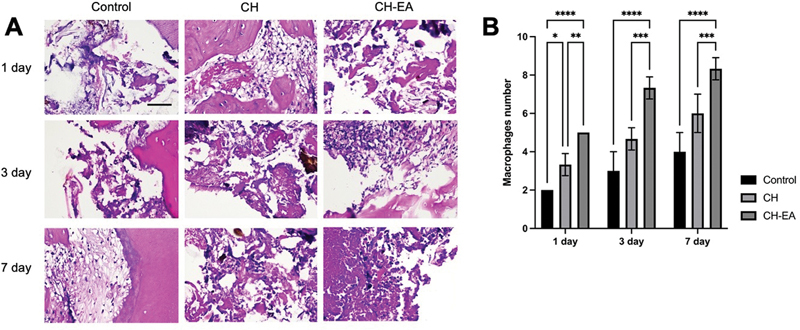
The histopathology section of dental pulp tissue with hematoxylin and eosin. ( **A** ) The macrophages appear at the magnification of 400x. ( **B** ) The comparison of macrophage numbers in each group. The application of calcium hydroxide and ellagic acid showed a higher number compared with the calcium hydroxide and control. The asterisk symbols indicate significant differences with using one-way and post hoc Tukey's honestly significant difference (HSD). * *p* < 0.05; ** *p* < 0.01; *** *p* < 0.001; **** *p* < 0.0001. CH, calcium hydroxide; CH-EA, calcium hydroxide-ellagic acid.

Conversely, pulp capping with calcium hydroxide and ellagic acid affects chronic inflammatory response by raising the number of macrophages after 1, 3, and 7 days compared with pulp capping with calcium hydroxide alone or the control group ( *p* < 0.05; Fig. 2 ).

### Lymphocytes

**Fig. 3:**
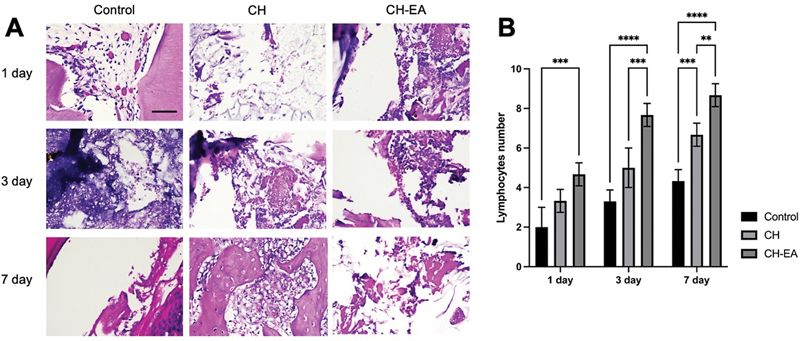
The histopathology section of dental pulp tissue with hematoxylin and eosin. ( **A** ) The lymphocytes appear at the magnification of 400x. ( **B** ) The comparison of lymphocyte numbers in each group. The application of calcium hydroxide and ellagic acid showed a higher number compared with the calcium hydroxide and control. The asterisk symbols indicate significant differences with using one-way and post hoc Tukey's honestly significant difference (HSD). ** *p* < 0.01; *** *p* < 0.001; **** *p* < 0.0001. CH, calcium hydroxide; CH-EA, calcium hydroxide-ellagic acid.

Similarly, pulp capping with calcium hydroxide and ellagic acid affects chronic inflammatory response by increasing the number of lymphocytes after 1, 3, and 7 days compared with pulp capping with calcium hydroxide alone or the control group ( *p* < 0.05; Fig. 3 ).

### Tumor Necrosis Factor-α Expression

**Fig. 4:**
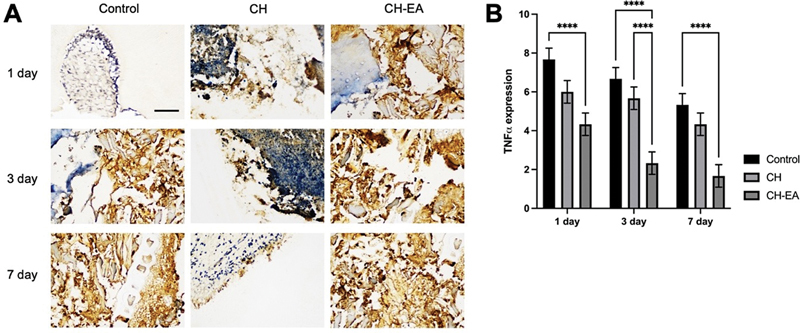
The immunohistochemistry staining of dental pulp tissue for localization of tumor necrosis factor-α TNF-α expression. ( **A** ) The TNF-α expression was analyzed at the magnification of 400x. ( **B** ) The comparison of TNF-α expression in each group. The application of calcium hydroxide and ellagic acid showed a lower expression compared with the control. The asterisk symbols indicated significant differences with using one-way and post hoc Tukey's honestly significant difference (HSD). **** *p* < 0.0001. CH, calcium hydroxide; CH-EA, calcium hydroxide-ellagic acid.

Despite the increase in macrophage numbers with calcium hydroxide and ellagic acid, TNF-α expression is reduced compared with calcium hydroxide and the control group at 1, 3, and 7 days ( Fig. 4 ).

## Discussion

The combination of ellagic acid with osteoinductive materials shows promising effects. Reported outcomes include the combination of ellagic acid with hydroxyapatite promoting bone regeneration by increasing the expression of several proteins related to angiogenesis, such as fibroblast growth factor 2 (FGF-2) and vascular endothelial growth factor (VEGF), 15 as well as proteins associated with osteogenesis, including alkaline phosphatase (ALP), 15 bone morphogenesis protein 4 (BMP4), 16 receptor activator of nuclear factor kappa-Β ligand (RANKL), 17 osteoprotegerin (OPG), 17 and osteocalcin (OCN) 17 and osteopontin (OPN). 16 Additionally, it has been shown to reduce inflammatory markers such as TNF-α and interleukin-10 (IL-10). 16

The idea is to propose the combination of calcium hydroxide and ellagic acid as pulp capping material because this combination has good physical characteristics and antibacterial properties. 18 In this research, the combination of calcium hydroxide and ellagic acid as a pulp capping material exhibits a dual effect on the inflammatory response in dental pulp. First, the combination decreases the acute inflammatory response by decreasing the neutrophils number. The inflammatory response that occurs in the dental pulp occurs due to mechanical trauma or is caused by calcium hydroxide itself. Second, the application of calcium hydroxide paste as pulp capping material can induce the formation of a necrotic layer due to the high pH level, accompanied by inflammation in the underlying tissue. The hydroxyl ions released by calcium hydroxide can trigger the activation of NF-κB, leading to an increase in proinflammatory cytokines. This results in enhanced neutrophil chemotaxis and increases reactive oxygen species (ROS), thereby activating the acute inflammatory process. 19 The mechanical trauma that is created in the teeth activates the inflammation response by the odontoblast cell. 20 The first response is by producing the ROS that led to acute inflammation. 21 With ellagic acid, the production of ROS can be inhibited and inhibit the neutrophils response in dental pulp. This occurs because ellagic acid has strong anti-inflammatory properties. 22

After controlling the acute inflammatory response, the combination of calcium hydroxide and ellagic acid showed a faster progression in the chronic inflammatory response, marked by an increase in the number of macrophages and lymphocytes. Both cells take over the neutrophils' function to perform phagocytosis. 23 Macrophages begin to phagocytose apoptotic neutrophils on the third day. Macrophages are the result of monocyte differentiation as they migrate from the blood vessels to the tissue, functioning as phagocytes and releasing collagenase, elastase, and proinflammatory cytokines. Macrophages also release platelet-derived growth factor (PDGF), which stimulates chemotaxis and proliferation of fibroblast, thereby accelerating wound healing. The activation of NFκB can increase the M1 phenotype as an inflammatory agent and decrease the phagocytic function of macrophages, thus preventing phenotype switching from M1 to M2. 24 Consequently, the dominant M1 macrophages produce a large amount of the proinflammatory cytokine TNF-α. 25 The increase in proinflammatory cytokines like TNF-α due to the activation of NFκB can lead to a prolonged inflammatory phase and alter the M2 phenotype, which acts as an inflammatory resolution agent and secretes growth factors.

The combination of calcium hydroxide and ellagic acid is a natural material that combines the acidic properties of ellagic acid with the basic pH of calcium hydroxide. Calcium hydroxide, which dissociates into Ca ^2+^ and OH ^–^ ions, 26 plays a role in enhancing cell proliferation and in the formation of dentin bridges. 27 The OH ^–^ ions from dissociated calcium hydroxide can increase the pH to around 12.5 to 12.8, causing mitochondrial dysfunction and increased cell respiration rates. Under these conditions, superoxide diffuses from the cytosol to the mitochondrial membrane, leading to mitochondrial de-energization or cell apoptosis and increased ROS. 28 Ellagic acid can mitigate the inflammation caused by calcium hydroxide by inhibiting the production of proinflammatory cytokines through the NFκB1 and HSP70 pathways, which strongly bind to toll-like receptors (TLRs), specifically TLR2 and TLR4. 29 Preliminary *in vitro* studies using fibroblast cells treated with the combination of calcium hydroxide and ellagic acid showed high cell viability and proliferation, 11 indicating that this combination material is nontoxic and can stimulate cell proliferation. The reduction of ROS by ellagic acid decreases the activation of the NFκB signaling pathway. 30 Consequently, the inhibition of NFκB signaling reduces IKK activation, resulting in the inhibition of IκBα degradation. This leads to a decrease in the expression of proinflammatory cytokines, including TNF-α.

In this study, it appears that macrophages were predominantly activated toward the M1 phenotype. This is evidenced by the low expression of TNF-α in the group treated with calcium hydroxide and ellagic acid. It seems that ellagic acid in this combination works by scavenging ROS, which are involved in the activation of NFκB. Ellagic acid possesses strong antioxidant properties that help in scavenging ROS, thereby indirectly inhibiting the activation of NFκB. The consequence of inhibiting NFκB activation is the reduced production of proinflammatory cytokines such as TNF-α, IL-6, and IL-1β, contributing to its anti-inflammatory effects. However, the study's limitations include the lack of analysis of macrophage phenotypes and the absence of the examination of other proinflammatory cytokines produced by M1 macrophages and growth factors produced by M2 macrophages. The other limitation is that there is no analysis of dentinal bridge observation. Therefore, further analysis is needed to determine the macrophage phenotypes and dentinal bridge formed after the administration of calcium hydroxide and ellagic acid.

Overall, the combination of calcium hydroxide and ellagic acid presents a promising advancement in dental materials, offering enhanced healing properties, reduced inflammation, and improved biocompatibility, potentially leading to better clinical outcomes and patient satisfaction. Further clinical studies and trials are necessary to fully establish its efficacy and safety in pulp capping application in humans.

## Conclusion

The combination of calcium hydroxide and ellagic acid as a pulp capping material exhibits a dual effect on the inflammatory response in dental pulp. While it effectively reduces the number of neutrophils, indicating a decrease in acute inflammation, it simultaneously increases the number of macrophages and lymphocytes, which are associated with chronic inflammation and tissue repair processes. Notably, despite the elevated presence of macrophages, TNF-α expression is reduced compared with the groups treated with calcium hydroxide alone or the control group. This suggests that the combination of calcium hydroxide and ellagic acid may modulate the inflammatory response in a complex manner, promoting a more controlled and potentially beneficial healing process.
